# Commentary: Effective communication about pregnancy, birth, lactation, breastfeeding and newborn care: the importance of sexed language

**DOI:** 10.3389/fgwh.2024.1519979

**Published:** 2025-01-15

**Authors:** Maddalena Giacomozzi, Maaike Muntinga, Sally Pezaro

**Affiliations:** ^1^ Department of Obstetrics and Gynaecology, Radboud University Medical Centre, Nijmegen, Netherlands; ^2^ Treat it Queer Foundation, Nijmegen, Netherlands; ^3^ Amsterdam University Medical Center, Amsterdam, Netherlands; ^4^ Research Centre for Healthcare and Communities, Coventry University, Coventry, United Kingdom

**Keywords:** gender equality, health equality, health disparities, perinatal care, sexual and reproductive health and rights (SRHR), inclusive language

**A commentary on**
Effective communication about pregnancy, birth, lactation, breastfeeding and newborn care: the importance of sexed language By Gribble KD, Bewley S, Bartick MC, Mathisen R, Walker S, Gamble J, Bergman NJ, Gupta A, Hocking JJ and Dahlen HG (2022). *Front Glob Womens Health*. 3:818856. doi: 10.3389/fgwh.2022.818856

## 1 Introduction

In “Effective Communication About Pregnancy, Birth, Lactation, Breastfeeding, and Newborn Care: The Importance of Sexed Language”, Gribble et al. (1) argue that incorporating “sexed language” into perinatal services addresses the needs of people with diverse gender identities, while “desexed language” purportedly disadvantages cisgender women. We agree with Gribble et al. (1) that effective communication is essential in promoting equitable perinatal care and advancing reproductive justice. However, we respectfully dispute their claim that “sexed language” promotes equitable perinatal care because it is based on scientific inaccuracies and misrepresentations. We also point out key areas where more thorough engagement with the literature leads to different conclusions. Finally, we note how their rhetorical strategies are harmful as they perpetuate the ongoing marginalization of women along with that of gender and sexual minorities.

### 1.1 Confusion about sexed and gendered language

Gribble et al. (1) dismiss gender-inclusive terminology such as “pregnant people” and argue for the (exclusive) use of the “sexed” term “woman” in perinatal care. However, using “woman” as an exclusively sexed term is misleading, because sexed terms refer to aspects of one's sex (e.g., female, male or intersex), while the concept of “womanhood” is shaped by social and cultural norms, expectations, and roles that vary across time and place. We concur that it is indeed crucial to specify how sex-related characteristics relate to health outcomes to avoid confusion. Yet it is for this exact reason why language must be precise and directly relevant to the characteristics being addressed (e.g., medical records detailing which organs are *in situ*), to prevent confusion.

The conceptualization of sex that Gribble et al. (1) refer to is outdated, often simplistic and unsupported by current scientific discourse. It is factually incorrect to assert that “only two gametes and pubertal pathways to adulthood and gamete production” exist, as numerous scientists have evidenced otherwise (2–6). Sex is multidimensional, encompassing at least three key elements (the 3Gs): genetics (chromosomal makeup), gonads (reproductive organs), and genitals (external sexual anatomy) (7). The constellation of people's 3G axis and one's biology is determinative of their ability to give birth, but their biological makeup does not determine whether they socially identify with the term “woman”—and likewise, neither does the social identity of women determine anyone's ability to give birth (7–9). Since the category of people who actually give birth is neither interchangeable with nor collapsible into any single gender identity, referring to birthing individuals as “women” is linguistically, socially, and scientifically inaccurate, and, to use the authors' own words, “imprecise”.

Gribble et al. (1) further explain gender as a socially constructed and culturally dependent phenomenon. However, they hyper-focus on gender identity as a subjective individualized experience and miss opportunities to operationalize gender beyond that. Specifically, they overlook the distinctions between gender norms, roles, relationships, and salience, and how these aspects, while idiosyncratic, are decisive in shaping individual health needs and barriers to perinatal services (10). By conflating sex with gender, the authors reproduce the factually misguided idea that only women give birth, and also that the ability to give birth is a prerequisite for womanhood. By doing so, they not only exclude all people who do not identify as women, but also reduce women's identities to their presumed ability to give birth. This further harms women and thwarts gender equity by reinforcing the oppressive patriarchal imperative that women shoulder all reproductive labour (11).

### 1.2 The risks of fallacious argumentation

Gribble et al. (1) employ several rhetorical strategies to persuade readers that gender-inclusive language is disadvantageous for cisgender women who need to access perinatal services. They assert that this has led to “decreasing overall inclusivity, dehumanizing; including people who should be excluded; being imprecise, inaccurate or misleading” (1). To support this claim, the authors adopt the “straw man fallacy”, which is the covert replacement of an original argument with a different, false proposition and the subsequent countering of that false claim as if it was the original (12). Moreover, the authors cite only a singular copy-paste error as evidence instead of grounding their reasoning in the broad, comprehensive and present-day literature (13).

The authors insist on the fact that gendered terms like “maternity” and “breastfeeding” have become contentious, often citing opinions rather than evidence to support this claim (14, 15). However, gendered terms are still widely and frequently used, and terms like “chestfeeding” are rare in contemporary perinatal services (16). This argumentative style reflects an oversimplification, problematization and exaggeration of gender-inclusive language use. This disconnect is problematic because it does not align with the experiences of perinatal service users (17).

Furthermore, the authors theorize that gender-inclusive language contributes to gender inequality and/or diverts attention away from it. However, they neglect to acknowledge the root cause of gender inequality: patriarchal oppression (18–22). Given that one of the central aims of gender-inclusive terminology is to mitigate patriarchy-driven health injustices, it is perplexing that Gribble et al. (1) portray gender-inclusive language as a direct threat to women's health whilst overlooking the contribution of patriarchal oppression to health inequalities.

Lastly, Gribble et al. (1) present the needs of cisgender women and those of trans and gender diverse (TGD) people as inherently oppositional and mutually exclusive by implying that accommodating the rights of one marginalized group (TGD people) necessarily restricts the rights of another (cisgender women). By suggesting that adopting gender-inclusive language “harms women and their children” (and even casually introducing unsubstantiated connections with domestic violence), the authors roll out a polarizing, harmful line of reasoning, pitting one marginalized group against another. The authors invoke medical ethics to emphasize the moral significance of these unfounded assertions, a rhetorical strategy which further highlights the flaws in their approach.

## 2 Discussion

Critiquing academic outputs such as Gribble et al. (1) is essential in fostering diverse and rigorous research. For a more comprehensive rebuttal grounded in the latest scientific literature, we direct readers to Pezaro et al. (11). We concur with Pezaro et al. (11) who understand the use of gender-inclusive language as a profoundly feminist endeavour, one that, by challenging the patriarchal structures that reinforce reproductive labour as being “women's work”, opens possibilities for optimal perinatal care and contributes to the collective advancement towards reproductive justice for all.
